# Supplementary material for: Sensitive and Stable Electrochemical Sensor for Folic Acid Determination Using a ZIF-67/AgNWs Nanocomposite
Source: Biosensors (Basel). 2022 May 31;12(6):382. doi: 10.3390/bios12060382 (PMC9221106; doi:10.3390/bios12060382)
Supplement: Supplementary file 1 [file biosensors-12-00382-s001.zip › biosensors-1737331-supplementary.pdf]

biosensors

MDPI

Sensitive and Stable Electrochemical Sensor for Folic Acid Determination Using a ZIF-67/AgNWs Nanocomposite

Yujiao Sun [1], Xue Wang [2] and Hao Zhang [1,2,*]

[1] Beijing Laboratory of Food Quality and Safety, College of Food Science and Nutritional Engineering, China Agricultural University, Beijing 100083, China; b20203060458@cau.edu.cn
[2] Department of Nutrition and Health, China Agricultural University, Beijing 100091, China; xwang326@cau.edu.cn
* Correspondence: zhanghaocau@cau.edu.cn; Tel./Fax: +86-10-62736344

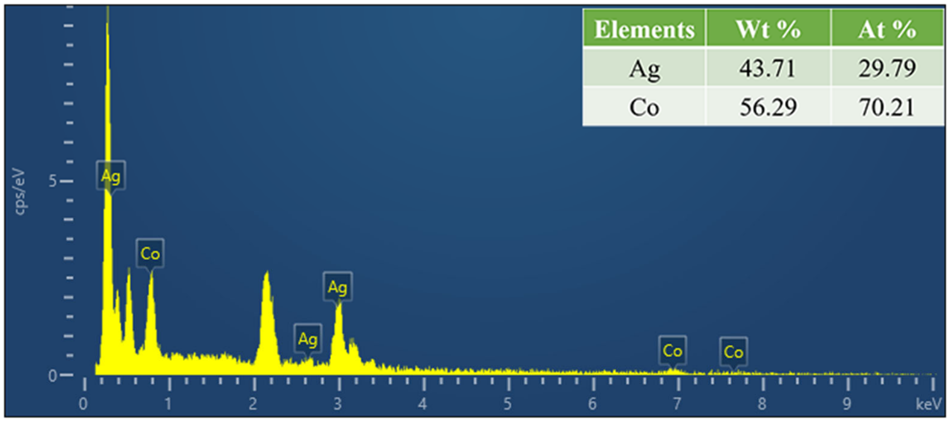

Figure S1. The EDS spectrum of ZIF-67/AgNWs.

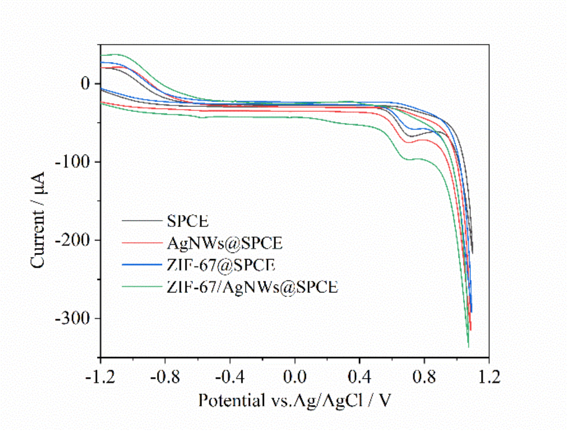

Figure S2. CV curves measured in the presence of FA (10 μM).

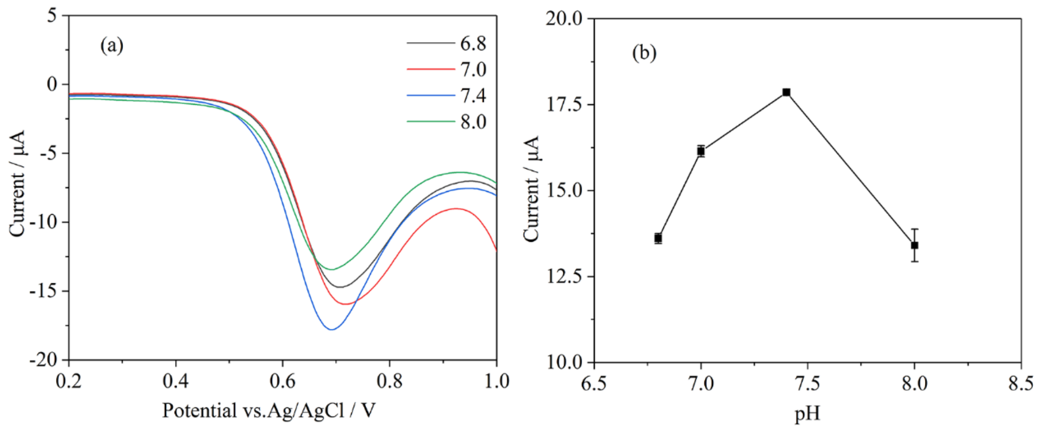

Figure S3. SWV curves of ZIF-67/AgNWs@SPCE in PBS with FA (10 μM) at different pH values (a). Relationship between pH and I_{FA} (b). All measurements were performed six times.

For the newly constructed ZIF-67/AgNWs@SPCE platform, the experimental pH was optimized via measurement of SWV curves. The modified SPCE was immersed in various pH values of PBS with FA (10 μM) added and the SWV curves were measured in different electrolyte solution. The pH of the electrolyte solution had a significant effect on the SWV curve measurements. With the pH increased, the SWV curve (I_{FA}) initially reduces and then increase, as shown in Figure S3a. The minimum of I_{FA} appears at 7.4 (Figure S3b). Based on the above experimental results, the optimal pH of the electrolyte solution for performing electrochemical signal measurements is 7.4 for ZIF-67/AgNWs@SPCE sensing platform.

Table S1. The value of different entities.

	**R_1 / Ω**	**R_2 / Ω**
SPCE	190.1	3406
AgNWs@SPCE	159.3	0.0041626
ZIF-67@SPCE	172.8	4032
ZIF-67/AgNWs@SPCE	175.1	0.0006749
